# Seroprevalence and associated risk factors of *Toxoplasma gondii* infection in domestic animals in southeastern South Africa

**DOI:** 10.4102/ojvr.v86i1.1688

**Published:** 2019-11-05

**Authors:** Whatmore M. Tagwireyi, Eric Etter, Luis Neves

**Affiliations:** 1 Department of Production Animal Studies, Faculty of Veterinary Science, University of Pretoria, Pretoria, South Africa; 2 CIRAD, UMR Animal, Santé, Territoires, Risque et Ecosystèmes (ASTRE), Montpellier, France; 3 ASTRE, Univ Montpellier, CIRAD, INRA, Montpellier, France; 4 Department of Veterinary Tropical Diseases, Faculty of Veterinary Science, University of Pretoria, Pretoria, South Africa; 5 Centro de Biotecnologia, Universidade Eduardo Mondlane, Maputo, Mozambique

**Keywords:** *Toxoplasma gondii*, latex agglutination test, seroprevalence, domestic animals, South Africa

## Abstract

*Toxoplasma gondii* is a major neglected parasitic infection occurring in settings of extreme poverty in Africa. Apart from causing reproductive failure in animals it is also a significant zoonotic concern. The objective of this study was to determine the seroprevalence and associated risk factors of *T. gondii* infection in cats, chickens, goats, sheep and pigs in the southeast of South Africa, of which little is known. Sera was obtained from 601 domestic animals including 109 cats, 137 chickens, 128 goats, 121 sheep and 106 pigs managed under different production systems in different agro-ecological regions and evaluated by the Toxoreagent, a latex agglutination test for *T. gondii* antibody detection. Household-level and animal-level data were collected by interviewing animal owners and/or herders using a closed-ended questionnaire. The study revealed an overall farm seroprevalence of 83.33% (125/150 farms) with the highest rate of infection for the parasite found in sheep with 64.46% (78/121), followed by goats with 53.91% (69/128), pigs with 33.96% (36/106), cats with 32.11% (35/109 cats) and chickens with 33.58% (46/137). The risk factors that were found to be statistically significant (*p* < 0.05) to different species of seropositivites were age, location, climate, animal production system, rodent control, seropositive cat, cat-feed access and cat faecal disposal. The relatively high seroprevalence of *T. gondii* detected in this region suggests that domestic animals may pose a substantial public health risk through the consumption of *T. gondii*-infected raw meat as well as via contact with cat faeces.

## Introduction

*Toxoplasma gondii*, a parasite with a wide range of mammalian and avian hosts, is the most successful and unrestricted parasitic pathogen (Torrey & Yolken 2013). Its definitive hosts are cats, which play an important role in the epidemiology of the parasite as the only species that sheds oocysts into the environment (Dubey 2009a, 2010). Humans and other warm-blooded animals are infected primarily by ingesting food or water contaminated with sporulated oocysts or by ingesting meat containing tissue cysts (Tenter, Heckeroth & Weiss 2000). Toxoplasmosis causes reproductive failure in animals, particularly in sheep, goats and pigs, resulting in huge economic losses (Abu Samra et al. 2007; Gebremedhin et al. 2015a). Apart from causing production losses in animals, toxoplasmosis is a significant zoonotic concern and can result in fatal disease such as encephalitis in immunocompromised people, abortions mainly in primiparous women infected during pregnancy and hydrocephalus in infants (Asgari et al. 2013; Dubey 2010). *Toxoplasma gondii* serological surveys have been conducted in both humans and animals in various parts of the world. However, in South Africa, literature on this is either out-dated or scant. Historical data concerning prevalence in humans reported by Mason, Jacobs and Flipp (1974) in the 1970’s revealed an alarming situation in South Africa, with up to 37.0% seroprevalence in some provinces in the then Transvaal (parts of Gauteng, Limpopo, North West, Mpumalanga and Kwazulu-Natal provinces) and a nationwide seroprevalence of 20.0% was detected a few years later (Jacobs & Manson 1978), demonstrating its importance as a zoonotic disease. There are very few references on domestic animals in South Africa, with 5.6% and 8.0% prevalence reported in sheep in 2007 and 2015 respectively and a prevalence of 37.1% in cats in 2015 (Abu Samra et al. 2007; Hammond-Aryee et al. 2015a; Hammond-Aryee, Van Helden & Van Helden 2015b). Toxoplasmosis-related illnesses have led to a surge in interest in the parasite, particularly with the onset of the current human immunodeficiency virus (HIV) epidemic (Hammond-Aryee, Esser & Van Helden 2014). Infected food-producing animals are considered to be the main sources of human infection and hence, the aim of the present study is to determine the seroprevalence of *T. gondii* infection and associated risk factors in food-producing animals and cats in the region.

## Materials and methods

### Study area

The study was conducted in all four local municipalities (Port St Johns/Nyadeni, Mhlontlo, King Sabata Dalindyebo and Ingquza Hill), in the Oliver Reginald District, in the Eastern Cape, South Africa, which covers a total area of 12 096 km^2^ and is located at 31° 34’ 00’’ S and 28° 46’ 00’’ E. There is no current data on the seroprevalence and associated risk factors across a wide array of domestic animals and the potential contribution each species plays to human infection in the district. Various factors within the Eastern Cape contribute to the need to assess the risk of toxoplasmosis within the district namely: the type of farming practised, the climatic conditions, informal slaughter and consumption of animals without meat inspection, the high HIV/AIDS prevelance and the harsh socio-economics of the region. The study area consists of mainly rural and peri-urban areas, which have a highly variable climate, mainly characterised by wet (subtropical) and arid (steppe) agro-ecological regions. It has one of the highest proportions of agricultural households without income (32.2%) and is the leading province in terms of livestock ownership (Lehohla 2013). The district accounts for 21.3% of the number of HIV positive people in the province, which has one of the highest HIV/AIDS prevalence rates, of 25.2% (19.8–31.5)_95%_, in the country (Human Sciences Research Council 2018).

### Sampling

A cross-sectional study design was conducted between June and October 2016 and venous blood samples were randomly collected from 601 domestic animals in 150 households from a target population of 278 250 indigent households (Eastern Cape Socio Economic Consultative Council 2017). The sample size determination for detection of disease for each of the five species under study was calculated using an expected disease prevalence threshold of 3% with a confidence level of 95%. The formula used for sample size determination is:

$$n=\frac{\log\alpha }{\log(1-P)}$$ [Eqn 1]

where *n* is the sample size, α is the accepted level of error (also considered as 1 – confidence) and *P* is the expected prevalence (Dohoo, Martin & Stryhn 2009). Thus, at least 100 samples were collected per species. To avoid any design effect resulting from intra-cluster correlation, only one sample from each species was taken from each household. Animals were randomly selected and the inclusion criteria for the different animal species sampled were animals present at routine community visits by veterinary officials during primary animal health campaigns. Samples were transferred to the laboratory on ice and following overnight refrigeration, serum was separated by centrifugation and stored at -20 °C in eppendorf tubes until analysis.

### Serology

All collected animal serum samples were tested for IgM/IgG antibodies against *T. gondii* using a commercial latex agglutination test, (Toxoreagent® RST701) according to the LAT manufacturer’s instructions (Toxoreagent®, Mast Group, United Kingdom). Agglutination at 1:64 or higher was regarded as positive except in chickens, where titres of 1:32 were regarded as positive (Hussien, Alfaki & Hussein 2016; Zia-Ali et al. 2007).

### Data collection and management

A pretested close ended questionnaire survey was conducted during blood sample collection by interviewing animal owners and/or herders to assess risk factors associated with toxoplasmosis. The hypothesized risk factors for *T. gondii* included location (municipality), type of climate (subtropical, steppe), sex (male, female), age (< 1 year, > 1 year), animal management system (extensive: free ranging without supplementary feed, semi-intensive: supplementary feed provided), biosecurity (no fence, one fence, double fence), source of drinking water (dam, river, borehole, tap), presence of cats (domestic and/ or feral), percentage of day spent on pasture (< 8 hours, > 8 hours, grazing distance from household (≤ 5 km, > 5 km), as well as presence or absence of rodent control, cat-feed contact, rodent-feed contact and rodent-animal contact.

The data generated were stored in an Excel spreadsheet and analysed using R software (R Core Team 2013). Maps were generated using the ‘RgoogleMaps’ package. Prevalence estimates were adjusted for a test sensitivity of 94.2% and a specificity of 96.6% reported by Holliman, Barker and Johnson (1990) in a study in humans, using the formula below:

$$TP=\frac{AP+Sp-1}{Se+Sp-1}$$ [Eqn 2]

where *TP* = true prevalence, *AP* = apparent prevalence, *Se* = test sensitivity and *Sp* = test specificity (Rogan & Gladen 1978:71–76). Confidence intervals (CI) were calculated using the following formula according to Dohoo et al. (2009):

$$CI=TP\pm Z_{\alpha /2}\sqrt{\frac{TP(1-TP)}{n}}$$ [Eqn 3]

where *TP* is the true prevalence, *n* the sample size and Z_α/2_ is the value of *Z* from the normal law for an accepted risk α (in our case α = 0.05 thus Z_*α*/2_ = 1.96). The Chi-Square test or the calculation of the odds ratio with a 95% confidence interval (CI) confirmed by the Fisher’s exact test were used to quantify the association between *T. gondii* seroprevalence and potential risk factors. The confidence level was set at 95% and the significance level was established at *p* < 0.05. The Moran autocorrelation coefficient was used to confirm the spatial risk assumed by seropositivity associated to specific municipalities, taking into account the spatial coordinates of the sampled households.

### Ethical considerations

The animal ethics committee for animal experimentation at the Faculty of Veterinary Science, University of Pretoria, South Africa, reviewed and approved the research proposal. Informed consent was obtained from animal owners and/or herders before collection of data and samples.

## Results

### Seroprevalence of *Toxoplasma gondii*

The study revealed a farm seropositivity of 83.33% (125/150) from which 40% of the seropositive farms were due to a single species. Antibodies to *T. gondii* were found in sheep (78/121) (58.89% – 75.61%)_95%_, in goats (69/128) (47.02% – 64.24%)_95%_, in pigs (36/106) (24.65% – 42.65%)_95%_, in chickens (46/137) (25.34% – 41.12%)_95%_ and in cats (35/109) (22.89% – 40.35%)_95%_ (Table 1).

**TABLE 1:** Seroprevalence of *Toxoplasma gondii* and statistically significant risk factors in domestic animals in southeastern South Africa (June 2016 – October 2016).

Species	Prevalence (%)		Risk factors	Chi-squared	Odds ratio	*p*
	True prevalence	95% CI				
Cat	31.62	22.89–40.35	Age	0.0075	3.43	0.0044
		2.12–17.75	Seropositive cat	-	5.77	0.0002
Chicken	33.23	25.34–41.12	Production system	-	2.90	0.015
Goat	55.63	47.02–64.24	Location	-	-	0.0014
Sheep	67.25	58.89–75.61	Age	0.0468	3.45	0.018
			Location	0.0186	-	-
			Cat-feed contact	0.0460	2.30	0.037
			Rodent control	0.0423	6.06	0.023
Pig	33.65	24.65–42.65	Climate	0.0077	3.37	0.0072
			Cat faeces covered with soil	0.0090	0.21	0.0085
			Cat faeces left in the environment	0.0198	5.20	0.0163

CI, confidence interval.

### Risk factors

**FIGURE 1:**
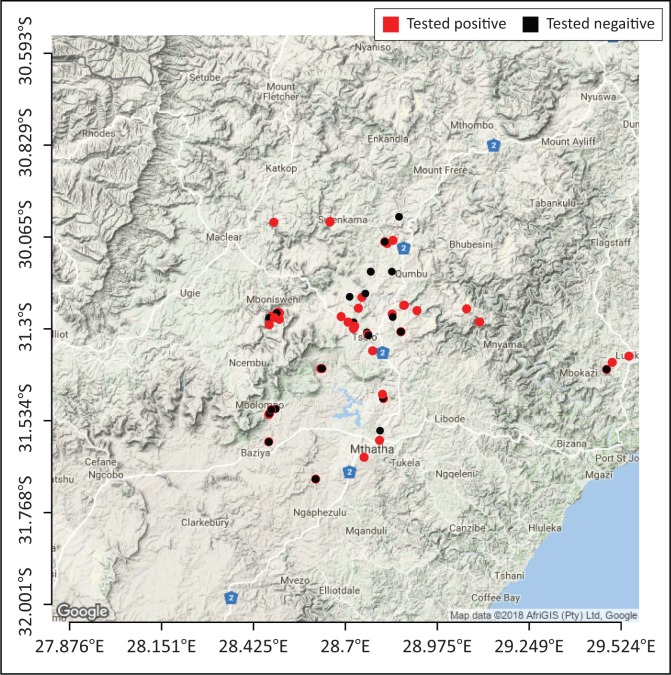
Geographic distribution of sampled households with sheep (red dots indicate positive test results and black dots indicate negative test results).

**FIGURE 2:**
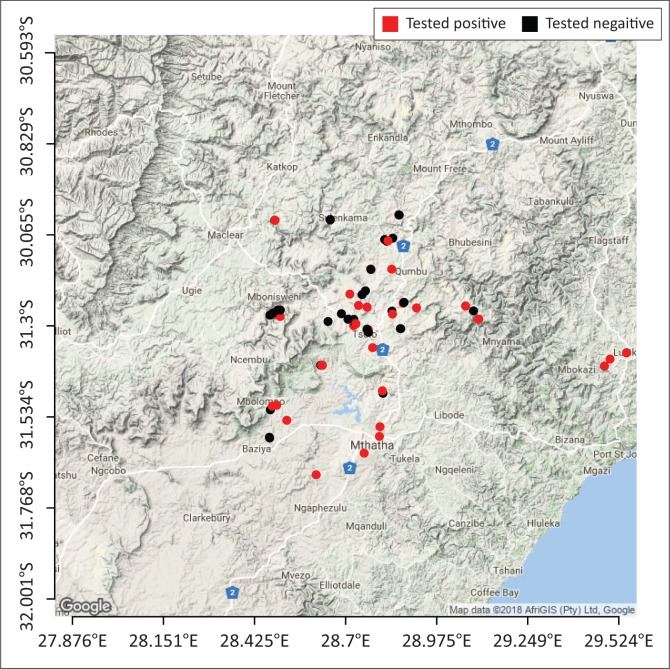
Geographic distribution of sampled households with goats (red dots indicate positive test results and black dots indicate negative test results).

Among the risk factors associated with increased *T. gondii* seropositivity for the different species, the following were found to be statistically significant: age, location, climate, animal production system, rodent control, seropositive cat, cat-feed access and cat faecal disposal (Table 1). Age in cats, was statistically significant with an odds ratio of 3.43, CI 95% (1.36–9.23) when comparing cats older and younger than 1 year. Seropositivity for *T. gondii* in cats increased significantly with age. The presence of a seropositive cat on a farm was a significant risk factor for seropositivity of the other animals on that farm. Seropositive cats increase the risk of having multiple species infection by 5.8 times CI 95% (2.12–17.75) as compared to seronegative cats. Chickens reared under intensive, small scale production systems were 2.9 times CI 95% (1.2–7.3) more likely to be seropositive for *T. gondii* infection as compared to those under extensive small scale conditions. Among the pigs, two risk factors were statistically significant; the type of climate and disposal of cat faeces. A significantly larger portion of the seropositive pigs (52.8%) were from steppe areas while 47.2% were from subtropical areas. Pigs in households that reported that cat faeces were covered up with soil were five times, odds ration (OR) = 0.21 CI 95% (0.06–0.72), less likely to test positive to the parasite when compared to those that reported that cat faeces were left in the environment. For sheep, the risk factors that showed significant association with an increased infection rate were location, age, cat-feed contact and rodent control. Sheep in the King Sabata Dalindyebo municipality had the highest seroprevalence (81.58%), followed by the Port St Johns municipality with 70% and lastly the Mhlontlo municipality with 54.79%. Older sheep had increased *T. gondii* seropositivity when compared to those less than one year of age. Additionally, households that reported cats having access to animal feed had a higher risk for sheep toxoplasmosis as compared to households that did not report cats having access to animal feed. *Toxoplasma gondii* infection in sheep was also higher in households that had rodent control than those that did not have any. For goats, the location was a statistically significant risk factor with the lowest *T. gondii* seroprevalence reported in the Mhlontlo municipality (43.75%), that of 63.16% in the King Sabata Dalindyebo municipality and the highest seroprevalence (100%) in the Port St Johns municipality. The Moran Index was significant for sheep and goats revealing a real spatial clustering of the positive cases (Figures 1 and 2).

## Discussion

### Farm seropositivity

The farm seroprevalence of 83.33% reported in this study is consistent with the observations of previous authors and supports the fact that the parasite has a wide distribution (Asgari et al. 2013; Dubey 2010). We observed that it is often the case that several species are infected with the parasite. The study showed that having a seropositive cat on a farm increased the risk of having multiple species infection as compared to having a seronegative cat. This supports the life cycle dynamics of *T. gondii* where cats act as definitive hosts and use warm-blooded animals as intermediate hosts as highlighted by Afonso et al. (2013). For this reason, infected domestic cats living in or around households will invariably expose the other species commonly found in the immediate vicinity to the parasite.

### Small ruminants

The high seroprevalence in small ruminants is similar to that obtained by Hove, Lind & Mukaratirwa (2005b) in Zimbabwe, where a seroprevalence of 67.7% and an eightfold difference in seroprevalence between sheep from rural areas (80%) and sheep from commercial farms (10%) was reported. This picture fits very well with that observed in this study, where almost all of the sheep sampled were from rural/semi-intensive farming systems and hence the nature of the sampling relative to the animal management system could have contributed to this. A similar trend has been found in numerous other studies where seroprevalence was much higher in sheep kept under intensive or semi-intensive management systems as opposed to those kept in extensive management systems (Abu Samra et al. 2007; Hove et al. 2005b; Van der Puije et al. 2000). However, the seroprevalence reported in sheep in this study is much higher than that of the two other studies conducted in sheep in South Africa by Abu Samra et al. (2007) and Hammond-Aryee et al. (2015b). This could be due to the differences in diagnostic methods used; the latex agglutination test used in this study is normally used as a screening test and has a comparably higher sensitivity than the indirect immune fluorescent test and the enzyme linked immune sorbent assay used in the other two studies (Holliman et al. 1990; Mazumder et al. 1988; Shaapan, Nawawi & Tawfik 2008). Another factor possibly contributing to this significant difference in seroprevalence is the geo-climatic conditions of the different regions in the studies. The Western Cape has a much colder minimum daily temperature range (8 °C – 14 °C) as compared to the Eastern Cape which has a much warmer minimum daily temperature range (14 °C – 17 °C) which is known to be favourable for oocyst sporulation (Abu Samra et al. 2007).

In the present study, it was observed that *Toxoplasma gondii* seroprevalence in sheep increased with age and other researchers have reported similar trends, where a higher seroprevalence were seen in ewes or rams than in lambs (Dubey 2009b; Dumètre et al. 2006; Gebremedhin et al. 2013). Households that reported cats having access to animal feed had a higher risk for toxoplasmosis in sheep as compared to households that did not report cats having access to animal feed. This finding is similar to that of other studies in which the presence of cats on farms increased seropositivity (Andrade et al. 2013; Dubey 2010). Seroprevalence of *T. gondii* in sheep was reported to be higher in households that had rodent control. This, however, is unusual as rodent control is thought to decrease *T. gondii* seropositivity (Kijlstra et al. 2008). Nevertheless, the high density of rodents in the sampled households may have instigated the implementation of rodent control measures, which were mostly biological (cats), increasing the likelihood of contamination of the environment by infective oocysts, which would put the sheep at risk (Dubey 2010; Lehmann et al. 2006; Opsteegh et al. 2012). Moran’s Index confirmed the seroprevalence differences between the municipalities, with Mhlontlo municipality showing the smallest seroprevalence for goats and sheep. Differences in the temperature and humidity of the three municipalities could have contributed to these variations in seroprevalence. The wet and humid climatic conditions of the district, particularly in the coastal areas, are conducive for oocysts sporulation and survival within the environment (Dubey et al. 1986; Van der Puije et al. 2000). The Mhlontlo municipality is entirely inland, while King Sabata Dalindyebo is partially coastal and Port St Johns is coastal.

### Cats

A seroprevalence of 31.62% reported in cats in this study is comparable to that of 37.1% reported in feral cats by Hammond-Aryee et al. (2015a) in the Western Cape. The similarity in the two reported prevalences could be attributed to the fact that domestic cats in rural areas behave like feral cats, as they are free to roam about, generally fending for themselves, hunting for food (rodents) and sometimes receiving leftovers from people. Cats older than one year were 3.43 times more likely to test positive for *T. gondii* as compared to those younger than one year. This trend is consistent with reports by other investigators, which supports the hypothesis of continuous exposure of cats to *T. gondii* oocysts in the environment with time (Afonso, Thulliez & Gilot-Fromont 2006; Ruiz & Frenkel 1980; Salant & Spira 2004).

### Pigs

The findings of this study revealed a seroprevalence of 33.65% in pigs, which is in agreement with those found in Ghana, Zimbabwe and Ethiopia, where researchers obtained seroprevalences within the range of 32.1% – 39% (Arko-Mensah et al. 2000; Gebremedhin et al. 2015a; Hove, Lind & Mukaratirwa 2005a). Similarities in the management of backyard pigs, which have access to infected swill, pasture and/or water contaminated with cat faeces could have contributed to these findings. Pigs in households that reported that cat faeces were covered up with soil were five times less likely to test positive for *T. gondii* when compared to those households that reported that cat faeces were left out in the open environment, allowing for much easier access. Though domestic cats tend to bury their faeces, which enhance survival of oocysts, burying them makes them inaccessible to other intermediate hosts, thereby reducing chances of infection, except for pigs that can burrow (Frenkel, Dubey & Miller 1970).

### Chickens

A seroprevalence of 33.23% was reported in chickens in this study and similar results were obtained in Ethiopia by Gebremedhin et al. (2015b) and in Nigeria by Ayinmode and Olaosebikan (2014), who reported prevalences of 30.5% and 40.4% respectively. The majority of chickens sampled in this study were free range and likely to have become infected when they fed on ground contaminated with oocysts. Backyard chickens under intensive small scale management systems showed higher seroprevalence since they have limited access to pasture and are confined to small areas, usually around households. These conditions are unhygienic and increase the probability of exposure to multiple sources of infective material (cat faeces, rodents, leftovers) as compared to extensively reared chickens, where the density of such infective material is lower.

## Conclusion

All species included in this study were exposed to *T. gondii*. Seroprevalences in the district varied from 31.62% – 67.25% depending on the sampled species. These convincingly show that the parasite is widely prevalent in the region and potentially causes reproductive failure in animals. Thus, it can be considered to be a significant zoonotic concern. In this study, we have determined the seroprevalence of *T. gondii* in sheep, goats, pigs, chickens and cats in the Oliver Reginald Tambo District. Additionally, age, geographical location, climate, rodent control, cat-feed access, cat faecal disposal seropositive cat and production system were found to be statistically significant risk factors of infection for animals in the district. This information on the epidemiological status of and risk factors associated with toxoplasmosis could help in the implementation of measures that could further reduce the burden of the disease in the district. Given the high seroprevalence of *T. gondii* in small ruminants, whose feeding patterns are different from those of the other species in the present study, it would be important for future research to examine the differences in the risk of infection between pastures and household surroundings.
